# Effects of patient beliefs regarding the need for antibiotics and prescribing outcomes on patient satisfaction in urgent-care settings

**DOI:** 10.1017/ash.2023.161

**Published:** 2023-04-26

**Authors:** Michael J. Cziner, Daniel E. Park, Rana F. Hamdy, Laura A. Rogers, Monique M. Turner, Cindy M. Liu

**Affiliations:** 1 Antibiotic Resistance Action Center, Department of Environmental and Occupational Health, Milken Institute School of Public Health, George Washington University, Washington, DC; 2 Division of Infectious Diseases, Children’s National Hospital, Washington, DC; 3 Department of Pediatrics, George Washington University School of Medicine and Health Sciences, Washington, DC; 4 Department of Communication, Michigan State University, East Lansing, Michigan

## Abstract

We studied how patient beliefs regarding the need for antibiotics, as measured by expectation scores, and antibiotic prescribing outcome affect patient satisfaction using data from 2,710 urgent-care visits. Satisfaction was affected by antibiotic prescribing among patients with medium–high expectation scores but not among patients with low expectation scores.

Antimicrobial resistance (AMR) affects the treatment of common diseases and is a growing public health threat. More than 2.8 million AMR infections occur in the United States each year, resulting in at least 35,000 deaths.^1^ AMR is accelerated by the overuse of antibiotics. Centers for Disease Control and Prevention (CDC) data from 2014 indicated that primary care providers (PCPs) in the United States accounted for 168 million oral antibiotic prescriptions, ∼63% of all oral antibiotic prescriptions administered.^2^ Approximately one-third of outpatient antibiotic prescriptions may be unnecessary.^3^ Providers who perceive that their patients expect antibiotics are more likely to prescribe antibiotics,^4^ which may be driven by the provider’s desire to maintain patient satisfaction. This factor may be especially applicable to urgent-care providers because they may fear that patients will seek care at competing urgent-care centers (UCCs). However, studies have not consistently shown that patients receiving an antibiotic prescription for an acute respiratory tract infection are more satisfied with their care.^5–8^ In this study, using patient questionnaires, we examined how antibiotic prescribing outcomes affect the association between self-reported patient beliefs regarding the need for antibiotics and patient satisfaction.

## Methods

In total, 29 general UCCs in Colorado, Florida, Georgia, and New Jersey and 5 pediatric UCCs in Texas participated in this study. At the conclusion of their visits, patients or guardians of pediatric patients were asked to complete an anonymous questionnaire about their demographics, expectation for antibiotics (expectation score), treatment plan, and level of satisfaction with their care (satisfaction score) (Supplementary Material 1). In the postvisit questionnaire, patient beliefs regarding the need for antibiotics were captured through a question about previsit expectations, “Did you think you would need an antibiotic before coming to urgent care?” to generate the expectation score. The questionnaire was provided in English, and questionnaires with incomplete answers for these questions were excluded from analysis.

The expectation score and the satisfaction score were measured using 5-point Likert scales, in which 5 represented the highest belief in the need for antibiotics or satisfaction. Associations between participant demographics, mean expectation score, and mean satisfaction score were assessed using analysis of variance through a general linear model. Relationships between demographic factors and antibiotic receipt were assessed using logistic regression. We fit a linear regression with an interaction term for antibiotic prescription and expectation score. Covariates selected for adjustment by backward selection included age, sex, race, ethnicity, and education. A mixed-effects model allowing clustering by clinic was conducted as a sensitivity analysis. Analyses were stratified by general and pediatric clinics. SAS version 9.4 software (SAS Institute, Cary, NC) was used for analyses.

## Results

Among 2,919 questionnaires returned from 27 general clinics and 546 returned from 5 pediatric clinics, 2,279 (78.1%) adult and 431 (78.9%) pediatric questionnaires were included for analysis after removing missing data (Table 1). There were more female compared with male survey respondents in both general clinics (n = 1,542 vs 737) and pediatric clinics (n = 372 vs 59). Most adult patients were aged 30–49 years (34.8%), followed by patients aged 50–64 years (25.0%) and patients aged 18–29 years (21.1%). Most adult patients were non-Hispanic (90.7%) and white (85.7%). Hispanic guardians of pediatric patients represented 29% of pediatric visits, and 73.5% were white. Nearly all adult patients (98.1%) graduated from high school, and 47.0% had a bachelor’s degree. Similarly, nearly all guardians of pediatric patients (97.2%) graduated from high school, and 49.9% had a bachelor’s degree.

**Table 1.** Demographics and Participant Characteristics for Adults at General Urgent-Care Clinics and Guardians of Pediatric Patients

Patient Characteristics	Antibiotics Prescribed/Participants, n/N (%)		Mean Expectation Score (SD)		Mean Satisfaction Score (SD)	
	Adult	Pediatric^a^	Adult	Pediatric^a^	Adult	Pediatric^a^
**Total**	1217/2279 (53.4)	155/431 (36.0)	3.37 (1.28)	3.26 (1.17)	4.23 (0.90)	4.18 (0.92)
**Sex**	†		†			
Male	363/737 (49.3)	18/59 (30.5)	3.26 (1.28)	3.07 (1.28)	4.18 0.88)	4.05 (0.97)
Female	854/1542 (55.4)	137/372 (36.8)	3.41 (1.27)	3.28 (1.15)	4.25 (0.90)	4.20 (0.91)
**Age group**	†		*		*	
18–29 y	232/482 (48.1)	45/123 (36.6)	3.44 (1.24)	3.22 (1.04)	4.14 (0.91)	4.21 (0.87)
30–49 y	410/794 (51.6)	92/212 (43.4)	3.31 (1.29)	3.45 (1.16)	4.22 (0.88)	4.25 (0.90)
50–64 y	319/569 (56.1)	15/78 (19.2)	3.36 (1.30)	2.79 (1.28)	4.29 (0.88)	3.96 (1.02)
≥65 y	256/434 (59.0)	3/18 (16.7)	3.39 (1.27)	3.17 (1.04)	4.25 (0.92)	4.22 (0.94)
**Ethnicity**						
Hispanic	114/211 (54.0)	50/125 (40.0)	3.40 (1.25)	3.38 (1.11)	4.16 (0.98)	4.18 (0.85)
Non-Hispanic	1103/2068 (53.3)	105/306 (34.3)	3.36 (1.28)	3.20 (1.19)	4.23 (0.89)	4.19 (0.95)
**Race**	†				†	
White	1069/1954 (54.7)	116/317 (36.6)	3.36 (1.28)	3.17 (1.19)	4.24 (0.89)	4.20 (0.90)
Black	57/130 (43.8)	11/36 (30.6)	3.52 (1.33)	3.53 (1.08)	4.27 (0.85)	4.14 (1.02)
Other or multiple	70/146 (47.9)	23/65 (35.4)	3.29 (1.21)	3.45 (1.09)	4.21 (0.90)	4.18 (0.86)
Chose not to share	21/49 (42.9)	5/13 (38.5)	3.31 (1.21)	3.69 (0.95)	3.73 (1.13)	3.85 (1.34)
**Education level**						
<HS diploma/GED	22/43 (51.2)	5/12 (41.7)	3.33 (1.25)	3.37 (0.97)	4.30 (0.74)	4.33 (0.78)
HS diploma/GED	368/689 (53.4)	50/114 (43.9)	3.44 (1.27)	3.34 (1.20)	4.20 (0.93)	4.11 (0.97)
Trade/associate’s degree	262/476 (55.0)	33/90 (36.7)	3.39 (1.29)	3.36 (1.05)	4.26 (0.87)	4.12 (0.90)
Bachelor’s degree	341/665 (51.3)	41/139 (29.5)	3.35 (1.28)	3.16 (1.22)	4.22 (0.89)	4.22 (0.93)
Master’s degree	152/286 (53.1)	20/58 (34.5)	3.19 (1.23)	3.31 (1.19)	4.19 (0.93)	4.31 (0.88)
Doctoral/professional degree	72/120 (60.0)	6/18 (33.3)	3.37 (1.32)	2.78 (1.16)	4.34 (0.80)	4.22 (0.88)

Note. SD, standard deviation.

† *P* < .05 among adult participants for a given characteristic.

* *P* < .05 among pediatric clinic participants for a given characteristic.

a Demographic and patient characteristics for guardians of pediatric patients.

Adult patient beliefs regarding the need for antibiotics, as measured by expectation scores for receiving antibiotics, did not differ significantly from guardians of pediatric patients: the mean expectation score of adults was 3.37 and the expectation score of guardians of pediatric patients was 3.26 (*P* = .095). Female adults had higher expectation scores compared to males: the mean expectation score of female patients was 3.41 and the mean expectation score of male patients was 3.26 (*P* = .009). Expectation scores did not differ by age, ethnicity, race, or education level for general clinics. For pediatric clinics, guardians aged 30–49 years had the highest expectation scores for antibiotic prescribing (expectation score, 3.45) compared with guardians aged 50–64 years (expectation score, 2.79; *P* < .001). Expectation scores did not differ by other characteristics.

More than half of adult patients were prescribed an antibiotic (53.4%). Female patients were significantly more likely to receive an antibiotic than male patients (55.4% vs 49.3%; *P* = .006). Prescribing increased with age; 48.1% of patients aged 18–29 years received an antibiotic, compared with 59.0% of patients ≥65 years (*P* = .004). White patients were more frequently prescribed antibiotics (54.7%) than were Black patients (43.9%) or patients reporting other or multiple races (48.0%; *P* = .02). There were no differences in prescribing patterns by ethnicity or education level.

Antibiotics were prescribed less frequently for pediatric visits (36.0%) than for adult visits (53.4%; *P* < .001). Guardians of pediatric patients aged 30–49 years were more likely to have antibiotics prescribed for their children (43.4%) than were guardians aged 50–64 years (19.2%) or guardians aged ≥65 years (16.7%; *P* < .001). Pediatric clinic prescribing patterns did not differ by sex, ethnicity, race, or education level.

Adult patients and guardians of pediatric patients indicated similar satisfaction scores: the satisfaction score for adults was 4.23, and the mean satisfaction score for guardians of pediatric patients was 4.18 (*P* = .372). Satisfaction scores were similar across sex, age group, ethnicity, and education level.

**Fig. 1.**
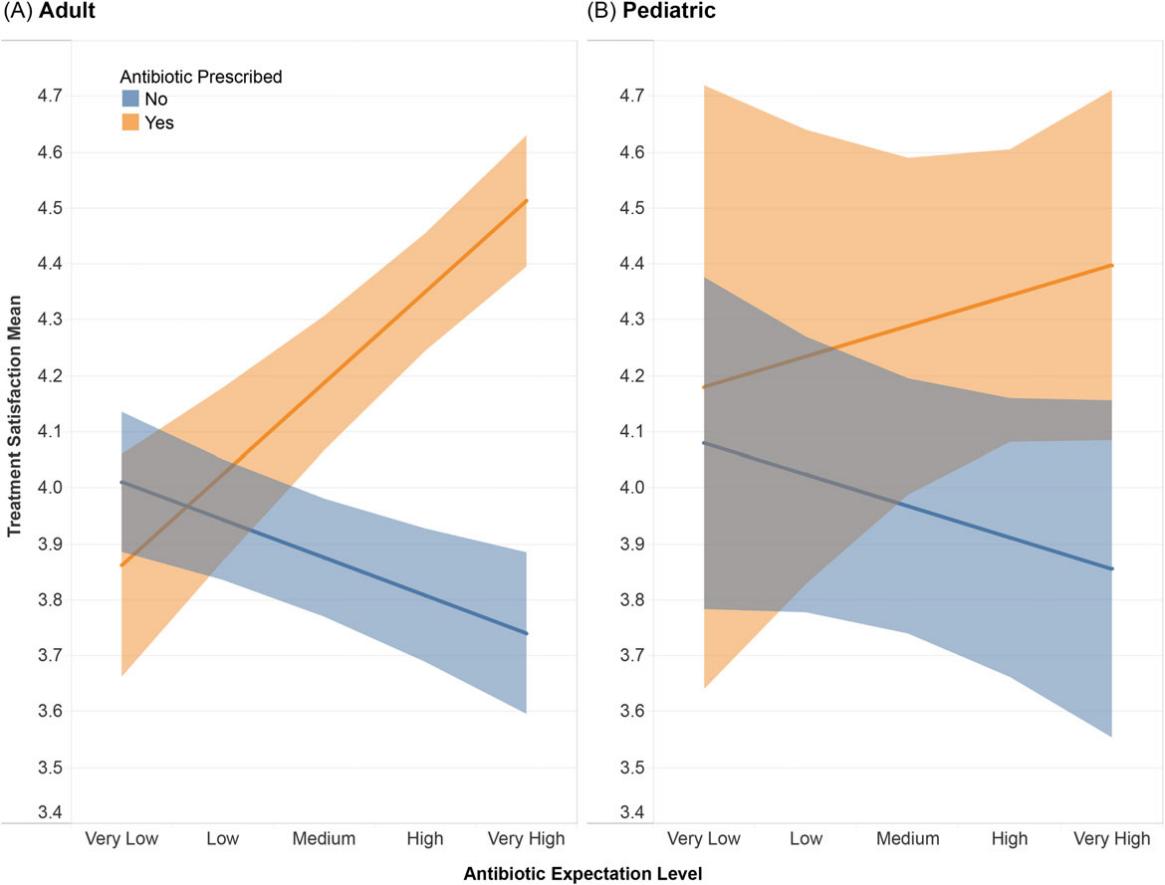
Treatment satisfaction scores by antibiotic expectation score and antibiotic prescription receipt for adult (A) and pediatric (B) patients. Individuals provided their satisfaction level to the treatment they received (1–5 Likert scale). Participants were grouped by their receipt of an antibiotic prescription and then by their expectation score for receiving an antibiotic. A statistical average was calculated for each group and is represented by the line in the shaded area. The shaded area represents 95% confidence intervals.

We evaluated whether antibiotic prescribing outcomes modify the association between patient satisfaction levels and patient beliefs regarding the need for antibiotics, as measured by expectation score for receiving antibiotics. Antibiotic prescription had no effect on patient satisfaction among patients reporting very low–to–low expectation scores. However, increasing expectation scores were associated with higher levels of satisfaction upon receiving antibiotics, and with lower levels of satisfaction without antibiotic prescription (adults_adjusted_ F = 42.4; *P* < .001) (Fig. 1). For pediatric visits, there was no statistically significant association (pediatrics_adjusted_ F = 1.3; *P* = .249).

## Discussion

Our study of patients from 32 UCCs in 5 states showed that antibiotic receipt greatly affected adult patient satisfaction with their care among patients self-reporting medium-to-very high expectation scores for receiving antibiotics, but antibiotic receipt did not affect treatment satisfaction when expectation scores were low.

Subgroup differences may provide insight into drivers of antibiotic prescription in urgent-care settings. White adult patients received antibiotics more frequently than Black adult patients; however, this disparity by race was not noted in children. Previous studies on antibiotic prescribing for viral upper respiratory infections and acute otitis media in children have shown differences in antibiotic prescribing by race. White children were more likely to receive antibiotics than Black children.^9,10^ Female adult patients were more likely to report beliefs for the need for antibiotics and to receive antibiotics than male patients. Future studies should explore contributing factors for these health disparities.

Our study had several limitations. There were fewer pediatric visits compared with adult visits; thus, while the pediatric interaction model resembled the adult model, it was likely underpowered to detect statistical significance. Antibiotic prescribing patterns may be affected by patient clinical history and provider expertise, which were not included in this analysis. For feasibility reasons, we did not have data to calculate the response rate and representativeness of the analytic population. There was high variability among the UCCs in patient response rates. Expectation scores were collected at the end of the visit and may be biased by patient education, prescribing outcomes, or other events during visit. Sensitivity analyses shifting patient expectations scores based on antibiotic receipt showed identical outcomes as the primary analysis. Additional sensitivity analyses included a mixed-effects model allowing random intercepts by clinic; results were similar to those of the primary analysis. Nonetheless, this study included a large geographically diverse sample among adults. The study spanned 32 clinics in 5 states, which represented unique population centers in various US regions and provide a broad and diverse sample of UCCs in the United States.

In this study, patient beliefs regarding the need for antibiotics were significantly associated with patient satisfaction only among patients with moderate-to-high expectation scores for antibiotics. However, previous studies have also shown that providers often incorrectly assume their patient’s expectations for receiving antibiotics. Our findings suggest that decreasing urgent-care patient expectations and beliefs regarding the need for antibiotics (eg, thoroughly educating patients on the clinical applicability and risks of antibiotic use) may decrease unnecessary prescriptions without negatively impacting patient satisfaction.

## Supplementary material

For supplementary material accompanying this paper visit http://doi.org/10.1017/ash.2023.161. click here to view supplementary material
