# Supplementary material for: Effects of patient beliefs regarding the need for antibiotics and prescribing outcomes on patient satisfaction in urgent-care settings
Source: Antimicrob Steward Healthc Epidemiol. 2023 Apr 26;3(1):e83. doi: 10.1017/ash.2023.161 (PMC10173279; doi:10.1017/ash.2023.161)
Supplement: Supplementary file 1 [file S2732494X23001614sup001.pdf]

SURVEY #1: MEDICAL DECISION MAKING FOR SELF

We invite you to participate in this questionnaire. Our clinic is participating in a randomized clinical trial on an important health issue. The survey should only take 5 minutes to complete. Please answer each question as honestly as possible. Your responses are completely anonymous. Responses to anonymous surveys cannot be traced back to the respondent. By taking this survey you are agreeing to be part of our study and allowing our team to use your anonymous data for research purposes. Your data will only be presented in aggregate form and No personally identifiable information is captured in this survey. Thank you.

First we would like to know a little about your experience today

1. Please take a look at the posters below. Which, if any, of these items did you see or receive **today**? Circle all that apply

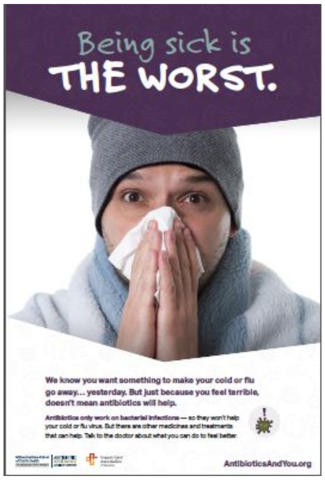

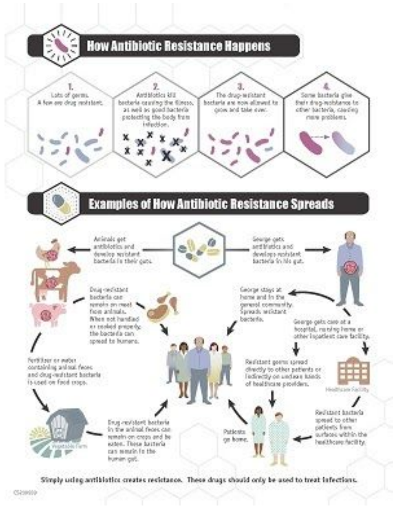

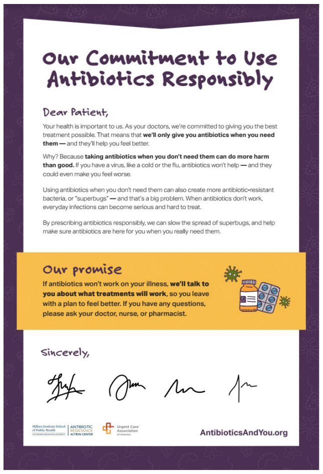

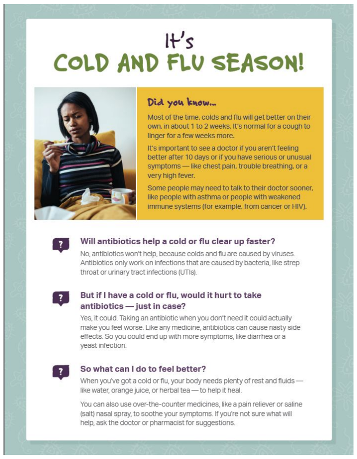

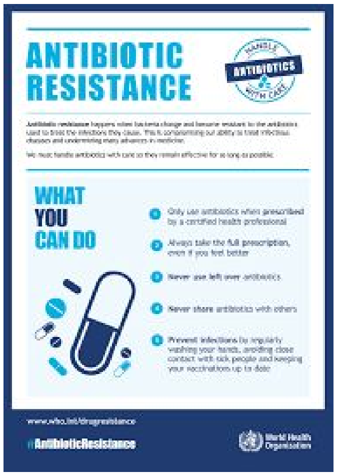

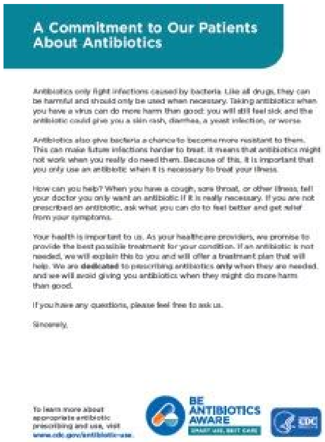

2. What was your main issue today and did you think you will need an antibiotic before coming to urgent care?
    - My main issue was ___________________________
    - I thought that I ___________________ need an antibiotic (circle one)
        - Definitely will
        - Probably will
        - Unsure
        - Probably won't
        - Definitely won't

3. Were you given any prescriptions today? (Circle all that apply)
    - Yes, antibiotics
    - Yes, other non-antibiotic prescriptions
    - Over-the-counter
    - No, I was not prescribed anything

4. Were you satisfied with this treatment plan? (circle one)
    a. Extremely satisfied
    b. Very satisfied
    c. Satisfied
    d. Less than satisfied
    e. Very unsatisfied

5. If you were expecting but did not receive an antibiotic today, what do you plan to do? (circle one)
    a. Follow the treatment plan recommended to me

b. I can use an over-the-counter treatment
c. I plan to go seek care elsewhere
d. Other: ____

We have some questions about how you think different infections should be treated

6. Which of these statements are accurate: (circle all that apply)
    a. Antibiotics will help a cold or flu clear up faster
    b. Antibiotics will help treat a viral infection
    c. Antibiotics will help to treat a bacterial infection
    d. None of the above

7. True/False: If a doctor is not sure if an antibiotic is needed, it is best if they prescribe one anyway, just in case
    a. True
    b. False
    c. I don't know

8. True/False: Antibiotic resistant infections are caused by bacteria "resistant" to certain antibiotics — meaning the antibiotics can't do their job anymore.
    a. True
    b. False
    c. I don't know

9. True/False: Antibiotic resistant infections are caused by bacteria "resistant" to certain antibiotics — meaning the antibiotics can't do their job anymore.
    a. True
    b. False
    c. I don't know

10. What can increase my risk for becoming infected by bacteria that are resistant to antibiotics?
    a. When I take an antibiotic and I become resistant to antibiotics
    b. When I take an antibiotic, which can cause bacteria to become resistant to antibiotics
    c. a and b are both correct
    d. I don't know

11. If you came to an urgent care clinic with any of the following medical conditions *would you expect* to receive an antibiotic? Please circle your answer.
    - Cold Yes/No

- Cough	Yes/No
- Virus	Yes/No
- Strep Throat	Yes/No
- Flu	Yes/No
- Urinary tract infection	Yes/No
- Bronchitis	Yes/No

Finally, we just have a few more questions about your background

12. How do you identify your biological sex/gender?
    - ❏ Male
    - ❏ Female
    - ❏ Other

13. What is your age?
    - ❏ 18-29 years old
    - ❏ 30-49 years old
    - ❏ 50-64 years old
    - ❏ 65 years or older

14. What is your ethnicity?
    - ❏ Hispanic or Latino
    - ❏ Non-Hispanic or non-Latino

15. How would you define your race (select all apply)
    - ❏ Caucasian / White
    - ❏ Black or African American
    - ❏ Native American or American Indian
    - ❏ Asian / Pacific Islander
    - ❏ Other ________________________
    - ❏ I choose not to answer

16. What is the highest degree or level of school you have completed? *If currently enrolled, highest degree received.*
    - ❏ No schooling completed
    - ❏ Nursery school to 8th grade
    - ❏ Some high school, no diploma
    - ❏ High school graduate, diploma or the equivalent (for example: GED)
    - ❏ Some college credit, no degree
    - ❏ Trade/technical/vocational training
    - ❏ Associate degree
    - ❏ Bachelor’s degree
    - ❏ Master’s degree
    - ❏ Professional degree
    - ❏ Doctorate degree

THANK YOU VERY MUCH FOR COMPLETING THIS QUESTIONNAIRE.

SURVEY #2: MEDICAL DECISION MAKING FOR OTHERS (E.G. PEDIATRICS)

We invite you to participate in this questionnaire. Our clinic is participating in a randomized clinical trial on an important health issue. The survey should only take 5 minutes to complete. Please answer each question as honestly as possible. Your responses are completely anonymous. Responses to anonymous surveys cannot be traced back to the respondent. By taking this survey you are agreeing to be part of our study and allowing our team to use your anonymous data for research purposes. Your data will only be presented in aggregate form and No personally identifiable information is captured in this survey. Thank you.

First we would like to know a little about your experience today

1. Which, if any, of these items did you see or receive today? Circle all that apply

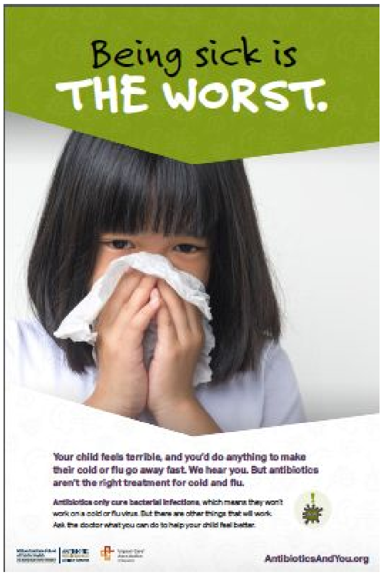

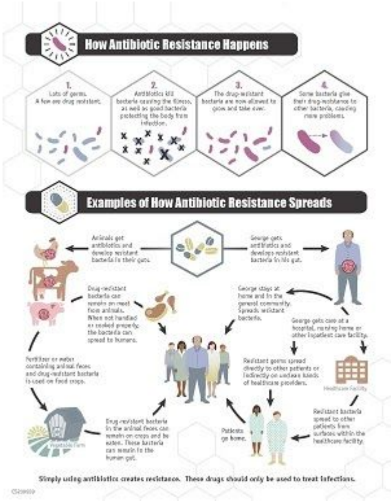

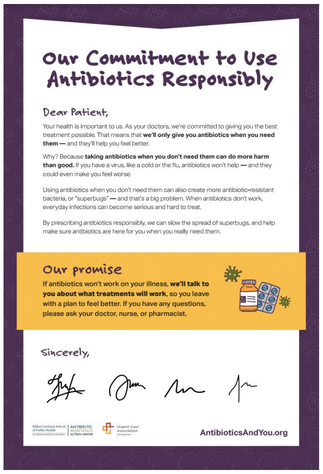

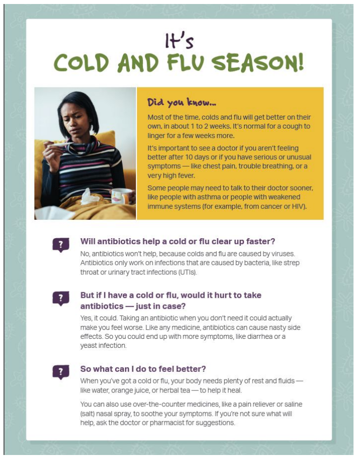

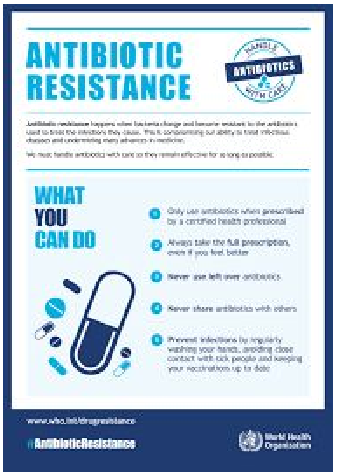

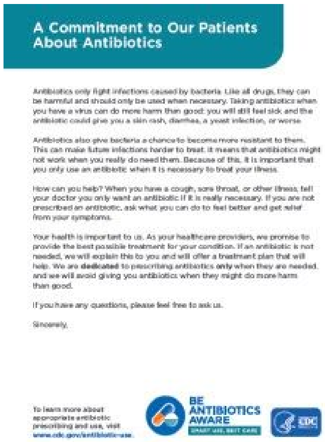

2. What was your child's main issue today and did you think she/he would need an antibiotic before coming to urgent care?
   - My child's main issue was ___________________________
   - I thought that my child ___________________ need an antibiotic (circle one)
     - Definitely would
     - Probably would
     - Unsure
     - Probably wouldn't
     - Definitely wouldn't

3. Was your child given any prescriptions today? (Circle all that apply)
   - Yes, antibiotics
   - Yes, other non-antibiotic prescriptions
   - Over-the-counter
   - No, I was not prescribed anything

4. Were you satisfied with the treatment plan recommended for your child? (circle one)
   a. Extremely satisfied
   b. Very satisfied
   c. Satisfied
   d. Less than satisfied
   e. Very unsatisfied

5. If you were expecting that your child would need an antibiotic, but did not receive one what do you plan to do? (circle one)
   f. Follow the treatment plan recommended for my child

g. I give my child an over-the-counter treatment
h. I plan to go seek care for my child elsewhere
i. Other: ____

We have some questions about how you think different infections should be treated

6. Which of these statements are accurate: (circle all that apply)
   j. Antibiotics will help a cold or flu clear up faster
   k. Antibiotics will help treat a viral infection
   l. Antibiotics will help to treat a bacterial infection
   m. None of the above

7. True/False: If a doctor is not sure if an antibiotic is needed, it is best if they prescribe one anyway, just in case
   e. True
   f. False
   g. I don't know

8. True/False: Antibiotic resistant infections are caused by bacteria "resistant" to certain antibiotics — meaning the antibiotics can't do their job anymore.
   n. True
   o. False
   p. I don't know

9. True/False: Antibiotic resistant infections are caused by bacteria "resistant" to certain antibiotics — meaning the antibiotics can't do their job anymore.
   q. True
   r. False
   s. I don't know

10. What can increase my risk for becoming infected by bacteria that are resistant to antibiotics?
   t. When I take an antibiotic and I become resistant to antibiotics
   u. When I take an antibiotic, which can cause bacteria to become resistant to antibiotics
   v. a and b are both correct
   h. I don't know

11. If you came to an urgent care clinic with any of the following medical conditions *would you expect* to receive an antibiotic? Please circle your answer.
   - Cold Yes/No

- Cough Yes/No
- Virus Yes/No
- Strep Throat Yes/No
- Flu Yes/No
- Urinary tract infection Yes/No
- Bronchitis Yes/No

Finally, we just have a few more questions about your background

12. How do you identify your biological sex/gender?
    - ❏ Male
    - ❏ Female
    - ❏ Other

13. What is your age?
    - ❏ 18-29 years old
    - ❏ 30-49 years old
    - ❏ 50-64 years old
    - ❏ 65 years or older

14. What is your ethnicity?
    - ❏ Hispanic or Latino
    - ❏ Non-Hispanic or non-Latino

15. How would you define your race (select all apply)
    - ❏ Caucasian / White
    - ❏ Black or African American
    - ❏ Native American or American Indian
    - ❏ Asian / Pacific Islander
    - ❏ Other ________________________
    - ❏ I choose not to answer

16. What is the highest degree or level of school you have completed? *If currently enrolled, highest degree received.*
    - ❏ No schooling completed
    - ❏ Nursery school to 8th grade
    - ❏ Some high school, no diploma
    - ❏ High school graduate, diploma or the equivalent (for example: GED)
    - ❏ Some college credit, no degree
    - ❏ Trade/technical/vocational training
    - ❏ Associate degree
    - ❏ Bachelor's degree
    - ❏ Master's degree
    - ❏ Professional degree
    - ❏ Doctorate degree

THANK YOU VERY MUCH FOR COMPLETING THIS QUESTIONNAIRE.
